# Changes in plasma cytokines following a 60‐h fast are not influenced by the addition of exercise despite elevated ketones in healthy young adults

**DOI:** 10.14814/phy2.70294

**Published:** 2025-03-25

**Authors:** Tori Bouck, Justin Monteleone, Jennifer Duffy, Philip N. Ainslie, Jonathan P. Little, Kate N. Thomas, Travis D. Gibbons, Hashim Islam

**Affiliations:** ^1^ School of Health and Exercise Sciences, The University of British Columbia Okanagan Kelowna British Columbia Canada; ^2^ Centre for Heart, Lung and Vascular Health School of Health and Exercise Sciences, The University of British Columbia Okanagan Kelowna British Columbia Canada; ^3^ Centre for Chronic Disease Prevention and Management, Faculty of Medicine The University of British Columbia Okanagan Kelowna British Columbia Canada; ^4^ Department of Surgical Sciences Dunedin School of Medicine, University of Otago Dunedin New Zealand; ^5^ Department of Biological Sciences Northern Arizona University Flagstaff Arizona USA

**Keywords:** immunometabolism, inflammation, interleukin‐10, interleukin‐6, leukocytes, tumor necrosis factor‐alpha

## Abstract

Immunometabolic processes maintain physiological homeostasis and are implicated in various chronic diseases. Fasting and exercise independently alter metabolic and immunological processes; their combination could provide insights into immunometabolic interactions. Using a randomized crossover design, 15 healthy adults (six females, nine males, 26.5 ± 4.3 years) fasted for 60 h with and without the addition of a 3 h cycling bout (65%–80% VO_2_ peak). Fasting alone (FAST) and with exercise (FEX) reduced plasma glucose, insulin, respiratory exchange ratio, and increased β‐hydroxybutyrate (BHB; all *p* < 0.01). FEX elicited more rapid changes in glucose and BHB and higher BHB concentrations at 60 h (all *p* < 0.01). Both conditions decreased circulating TNF‐⍺ concentrations and increased IL‐10 (*p* < 0.01), although the increase in IL‐10 appeared to be driven by the FEX condition (*p* = 0.03). IL‐6 concentrations tended to increase in both conditions (*p* = 0.1). Total white blood cell count remained unchanged after 60 h in both conditions, with only modest changes in some leukocyte subpopulations. Collectively, the observed changes in circulating cytokine concentrations support an overall anti‐inflammatory effect of prolonged fasting, while the maintenance of leukocyte concentrations suggests immune function is not compromised. Despite greater metabolic strain, the addition of prolonged exercise did not appear to augment changes in systemic cytokines and leukocytes.

## 1 INTRODUCTION

Interactions between inflammatory and metabolic processes—collectively referred to as immunometabolism—are critical for maintaining normal physiological function (Mathis & Shoelson, 2011). Impairments in insulin sensitivity and fatty acid oxidation characteristic of metabolic disorders contribute to the activation of pro‐inflammatory pathways, which in turn further disrupts metabolic processes (Smith et al., 2018). Interrogating this complex reciprocal relationship between immunological and metabolic defects—which are implicated in the most common chronic diseases (e.g., cardiovascular disease, diabetes, cancer, and dementia)—could advance our mechanistic understanding of immunometabolism. This knowledge could pave the way for potential therapeutics for chronic diseases or optimize healthy lifestyle recommendations. Although research from preclinical models has allowed mechanistic insight into immunometabolic interactions in response to different physiological stimuli or chronic disease states (Hotamisligil, 2017), comparatively little is known about the mechanistic basis of immunometabolic processes in healthy humans.

Fasting has gained popularity in recent years due to its purported widespread health benefits, and growing evidence suggests that fasting may improve immunometabolic processes (Marko et al., 2024). From a metabolic perspective, fasting shifts fuel selection by enhancing fatty acid oxidation and decreasing reliance on carbohydrates (Anton et al., 2018). Such alterations in substrate utilization may be linked to changes in immunological processes; for instance, the upregulation of fatty acid oxidation could favor the polarization of macrophages to a more anti‐inflammatory phenotype, which relies more heavily on oxidative metabolism through the breakdown of fatty acids (Odegaard & Chawla, 2011). On the other hand, fasting‐induced reductions in glucose oxidation could limit the development of a pro‐inflammatory macrophage phenotype, which relies heavily on glycolytic (i.e., carbohydrate) metabolism (Ménégaut et al., 2019). Prolonged fasting also promotes the production of ketone bodies, which serve as an alternative fuel source to maintain cellular processes as hepatic and muscle glycogen stores become depleted (Newman & Verdin, 2017). The ketone body β‐hydroxybutyrate (BHB) may exert anti‐inflammatory effects through inhibition of the NOD‐like receptor protein 3 (NLRP3) inflammasome and associated release of pro‐inflammatory cytokines (Youm et al., 2015), and increased secretion of anti‐inflammatory cytokines (e.g., IL‐10) (Neudorf et al., 2024). Exposure to endogenous BHB may therefore present another mechanism explaining the immunological and inflammatory effects associated with prolonged fasting. Accordingly, various fasting protocols have been shown to induce anti‐inflammatory effects, as demonstrated by reduced circulating pro‐inflammatory cytokines (Wang et al., 2020; Zouhal et al., 2020), greater anti‐inflammatory cytokine secretion (Janssen et al., 2023; Jordan et al., 2019) as well as reductions in C‐reactive protein (Madkour et al., 2023; Wang et al., 2020) and circulating lymphocytes (Fazeli et al., 2020) and monocytes (Collins et al., 2019; Janssen et al., 2023). However, given the significant heterogeneity in the type and duration of fasting protocols implemented, as well as the lack of in vivo mechanistic studies conducted in humans, further research is warranted to determine the immunological effects of an acute prolonged fast in humans. There is also a need to explore the interaction between immunological processes and fasting‐induced alterations in whole‐body metabolism to advance our understanding of immunometabolic responses to physiological stress.

Like fasting, exercise also influences both metabolic and immunological processes (Rosa‐Neto et al., 2022), and thus presents another physiological stimulus to study the relationship between whole‐body metabolism and immune function. Acute exercise is widely accepted to promote anti‐inflammatory effects, most notably through the contraction‐mediated release of IL‐6 from skeletal muscle (Petersen & Pedersen, 2005) and secretion of adrenal hormones (Pedersen & Hoffman‐Goetz, 2000). These changes subsequently lead to an increase in anti‐inflammatory cytokine secretion, which can downregulate the release of pro‐inflammatory cytokines (Gleeson et al., 2011). Additionally, as acute exercise increases energy expenditure, fatty acid oxidation, and muscle glycogen utilization (Goodpaster & Sparks, 2017), it seems reasonable to speculate that exercise‐induced alterations in both metabolic and immune processes could compound the immunometabolic effects of a subsequent fast, and/or amplify the immunological effects of fasting due to increased metabolic strain. Although the combined effects of fasting and exercise on immunometabolism remain understudied in humans, the combination of these two physiological stimuli provides an attractive model to study the relationship between altered whole‐body metabolism and corresponding changes in immunological and inflammatory processes.

Therefore, the purpose of the present study was to determine the effects of a 60‐h fast—with or without the addition of a prolonged exercise bout following the first 12 h of fasting—on immunological and inflammatory processes in healthy adults. We hypothesized that prolonged fasting would lead to a more anti‐inflammatory circulating cytokine profile, which would be augmented by the addition of a prolonged exercise bout after 12 h of fasting. This model allowed us to explore the interaction between alterations in whole‐body metabolism and immune function while also furthering our understanding surrounding the immunomodulatory effects of fasting and the potential for prolonged exercise to augment these effects.

## 2 METHODS

### 2.1 Participants

Fifteen healthy young adults were recruited based on the following inclusion criteria: (1) 18–40 years of age, (2) cleared to engage in exercise as indicated by the Physical Activity Readiness Questionnaire (PARQ+), and (3) accustomed to performing high‐intensity aerobic exercise. Participants were excluded if they: (1) had a history of cardiometabolic diseases (e.g., diabetes and cardiovascular disease), (2) had a history of smoking, (3) had a history of diagnosed or undiagnosed eating disorders, and (4) were taking medication (with the exception of oral contraceptives). Verbal and written informed consent was obtained from all participants prior to data collection in accordance with the Declaration of Helsinki, and the study was approved by UBC's clinical research ethics board (H22‐01713). All data collection was performed at the University of British Columbia (Okanagan) in Kelowna, BC, Canada.

### 2.2 Experimental design

A randomized crossover design was implemented, with participants performing a 60‐h fast on two separate occasions separated by a washout period of at least 3 weeks. In one condition, participants performed an exercise protocol after 12 h of fasting (FEX), whereas in the alternate condition participants abstained from exercise throughout the duration of the 60‐h fast (FAST). In each experimental condition, participants arrived at the lab following a 12‐h overnight fast, after which a blood sample was taken to obtain baseline measures for hematology, plasma cytokines (interleukin [IL]‐6, IL‐10, and tumor necrosis factor‐alpha [TNF‐⍺]), and metabolic markers (e.g., beta‐hydroxybutyrate; BHB, glucose, and insulin), and resting gas exchange was assessed using indirect calorimetry. Participants were given continuous glucose monitors (Freestyle Libre Pro 2) to wear throughout the intervention, and the fast was terminated if blood glucose levels dropped below 2.5 mmol/L. In the FEX condition, participants then completed 3 h of cycling targeting 65%–80% of their individual VO_2_ max (as determined by a prior maximal exercise aerobic test on a cycle ergometer). All participants completed a 3‐h familiarization cycle session to identify the maximum workload that could be achieved without fuel supplementation. Three‐minute breaks were given at 60‐ 120‐, and 150‐min, and participants were given 600–900 mL/hour of zero‐calorie sweetened isotonic beverage (Splenda™) to maintain euhydration. In the FAST condition, no exercise was performed at this time point. Participants were then instructed not to consume any calories throughout the duration of the fast but were allowed to consume black coffee or tea. Participants were asked to match caffeine intake before all testing days and to replicate caffeine consumption to the best of their abilities between conditions. Blood samples and respired gases were collected again after 36 and 60 h of fasting in each condition.

### 2.3 Blood sample collection and analysis

Venous blood samples were taken at 12, 36, and 60 h of fasting in each condition (4 mL × 2 EDTA tubes, plasma; 4 mL in one SST tube, serum; 12 mL total). One EDTA tube was placed at 4°C until complete blood counts could be analyzed (<24 h) using a hematology analyzer (Beckman DxH520). The remaining EDTA tube was centrifuged immediately (15 min, 2600 RCF at 4°C). Plasma was carefully removed and frozen at −80°C for batch analysis. Circulating metabolic markers (glucose, BHB, lactate, and insulin) were measured in serum using a Cobas 8000 Analyser (Roche Diagnostics). Plasma cytokine concentrations (IL‐10, IL‐6, and TNF‐⍺) were determined using a high‐sensitivity multiplex assay (V‐plex, MesoScale Discovery, catalogue number: K15049D) according to manufacturer instructions. Samples were diluted 1:1 according to the specifications of the kit and batch analyzed on a MESO QuickPlex SQ 120 Imager (Mesoscale Discovery).

### 2.4 Respiratory gas exchange

Respiratory gas exchange was measured at 12 h, 36 h, and 60 h in each experimental condition to assess resting metabolic rate, as well as the relative contribution of fat and carbohydrates to energy production at each timepoint (as measured by the respiratory exchange ratio; RER). Briefly, participants remained supine for 10 min while respired gases were sampled at the mouth. Respiratory variables were acquired using a metabolic cart (COSMED Quark, CPET + RMR, Roma, Italy).

### 2.5 Statistical analysis

Data were analyzed in RStudio. Baseline characteristics are presented as mean ± SD. Viable data from all participants were included in an intention‐to‐treat analysis; however, statistical outliers were removed using interquartile range with a multiplier of 2.2 (Pedersen & Hoffman‐Goetz, 2000). Briefly, the interquartile range was calculated and multiplied by 2.2. This value was added to the 75th percentile to determine the upper threshold and subtracted from the 25th percentile to determine the lower threshold. Data points that fell outside of this range were removed from the analysis. All cytokine data for one participant during FEX were removed due to abnormally high circulating levels of IL‐6, IL‐10, and TNF‐⍺. Similarly, IL‐6 levels for one participant at 60 h were abnormally elevated and thus removed from analysis. TNF‐⍺ data from one participant was removed in FAST due to very low circulating levels. Two participants exhibited abnormally high RMR values (one in FAST condition and one in FEX)—potentially due to measurement error attributable to limited duration of gas exchange and lack of a prior rest period—and were therefore removed from analysis. Data were analyzed using linear mixed effects models with condition (FAST vs. FEX), time point (12 h vs. 36 h vs. 60 h), and the interaction between condition and time point as fixed effects, and a random intercept for participant. Linear regression analysis was used to assess relationships between metabolic and inflammatory outcomes. Normality was assessed through visual inspection of Q‐Q plots and Shapiro–Wilk tests. Outcome variables that deviated significantly from a normal distribution were log‐transformed where appropriate. Significance was set at *p* < 0.05. Significant main effects of time and condition*time interactions were followed up with preplanned pairwise comparisons within conditions post hoc.

## 3 RESULTS

### 3.1 Participant characteristics

Participant characteristics are shown in Table 1. All participants completed the study with 100% adherence to the experimental protocol, with the exception of one dropout in the FEX condition. Viable data for circulating IL‐6 was not obtained for one participant, and statistical outliers were removed as described above.

**TABLE 1:** Participant characteristics (*n* = 15; 6 females/9 males).

Age (years)	26.5 (4.3)
Height (cm)	176.7 (9.6)
Weight (kg)	70.9 (9.4)
Body Mass Index (kg/m^2^)	22.6 (1.6)
VO_2peak_ (mL/kg/min)	51.0 (8.2)

*Note*: Values are mean (standard deviation).

### 3.2 Whole body metabolic markers

**FIGURE 1:**
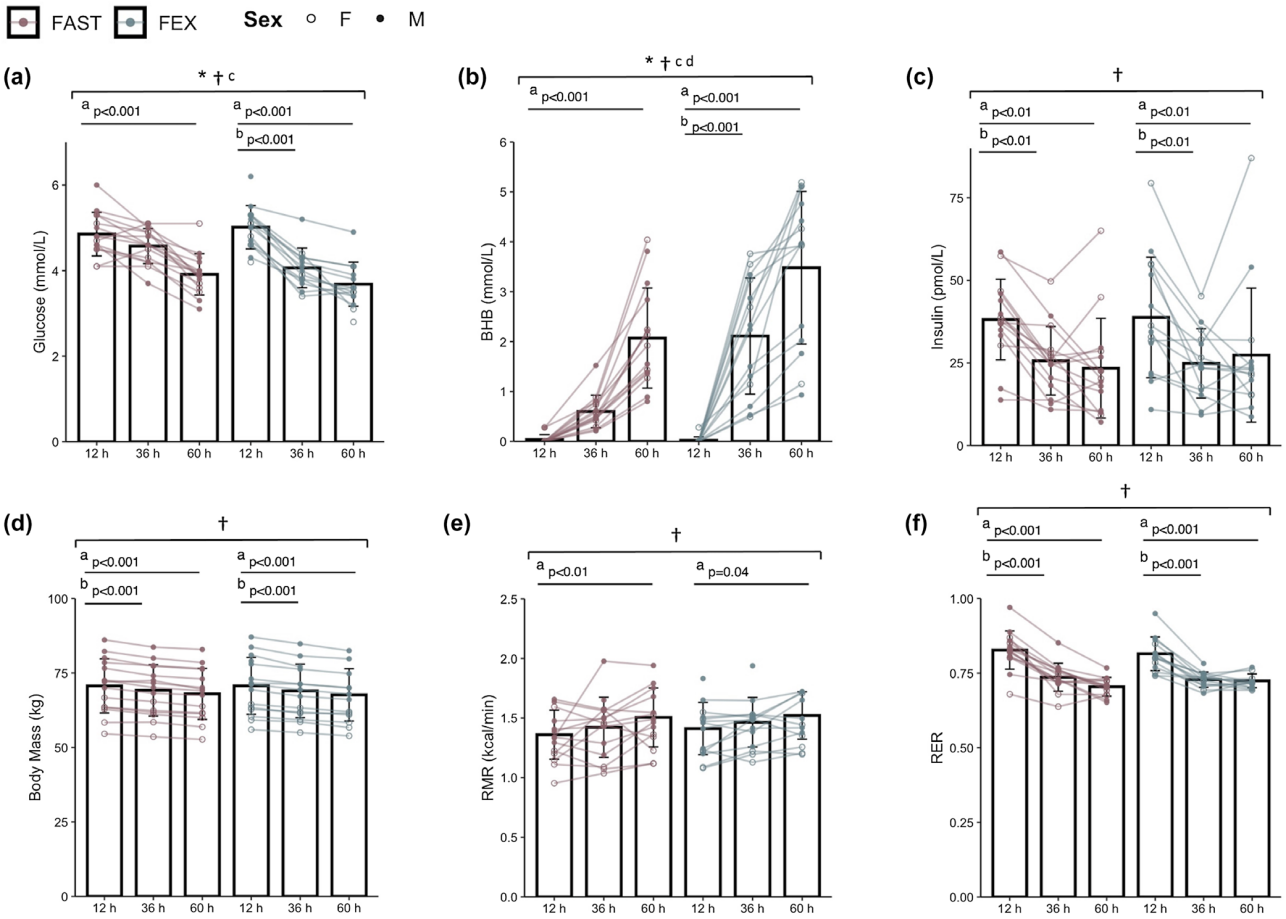
Changes in blood glucose (a), β‐hydroxybutyrate (BHB; (b), insulin (c), body mass (d), resting metabolic rate (RMR; e), and respiratory exchange ratio (RER; f) during each experimental condition. * Condition × Time Interaction, *p* < 0.05; ^†^Main effect of time, *p* < 0.05; ^a^12 h versus 60 h, *p* < 0.05; ^b^12 h versus 36 h, *p* < 0.05; ^c^FAST versus FEX at 36 h, *p* < 0.05; ^d^FAST versus FEX at 60 h, *p* < 0.05. Data are disaggregated by sex, with open circles (_°_) indicating female participants and closed circles (•) indicating male participants.

Changes in body mass, plasma glucose, insulin, beta‐hydroxybutyrate (BHB), resting metabolic rate (RMR), and RER are shown in Figure 1a–f. Significant main effects of time were observed for plasma glucose, BHB, RER, RMR, and body mass (all *p* < 0.001), and significant condition*time interactions were observed for BHB and plasma glucose (*p* < 0.001). Plasma glucose decreased from baseline to 60 h in both conditions (*p* < 0.001); however, a statistically significant decrease at 36 h was only observed in FEX (*p* < 0.001). Insulin was significantly decreased at 36 h in both conditions (both *p* < 0.01), with no further decrease after 60 h. BHB was significantly increased from baseline to 60 h in both conditions (*p* < 0.001), with a more rapid increase from baseline to 36 h observed in FEX (*p* < 0.001). BHB was significantly higher at 60 h in FEX than FAST (*p* < 0.001). RER was lower at 36 h and 60 h relative to baseline in both FAST and FEX (both *p* < 0.001). RMR was significantly increased after 60 h in both conditions (FAST; *p* < 0.01, FEX; *p* = 0.04), with no observable changes relative to baseline following 36 h of fasting. Body mass was significantly decreased after 36 h (*p* < 0.001), with a further decrease at 60 h (*p* < 0.01) in both conditions.

### 3.3 White blood cells and platelets

**FIGURE 2:**
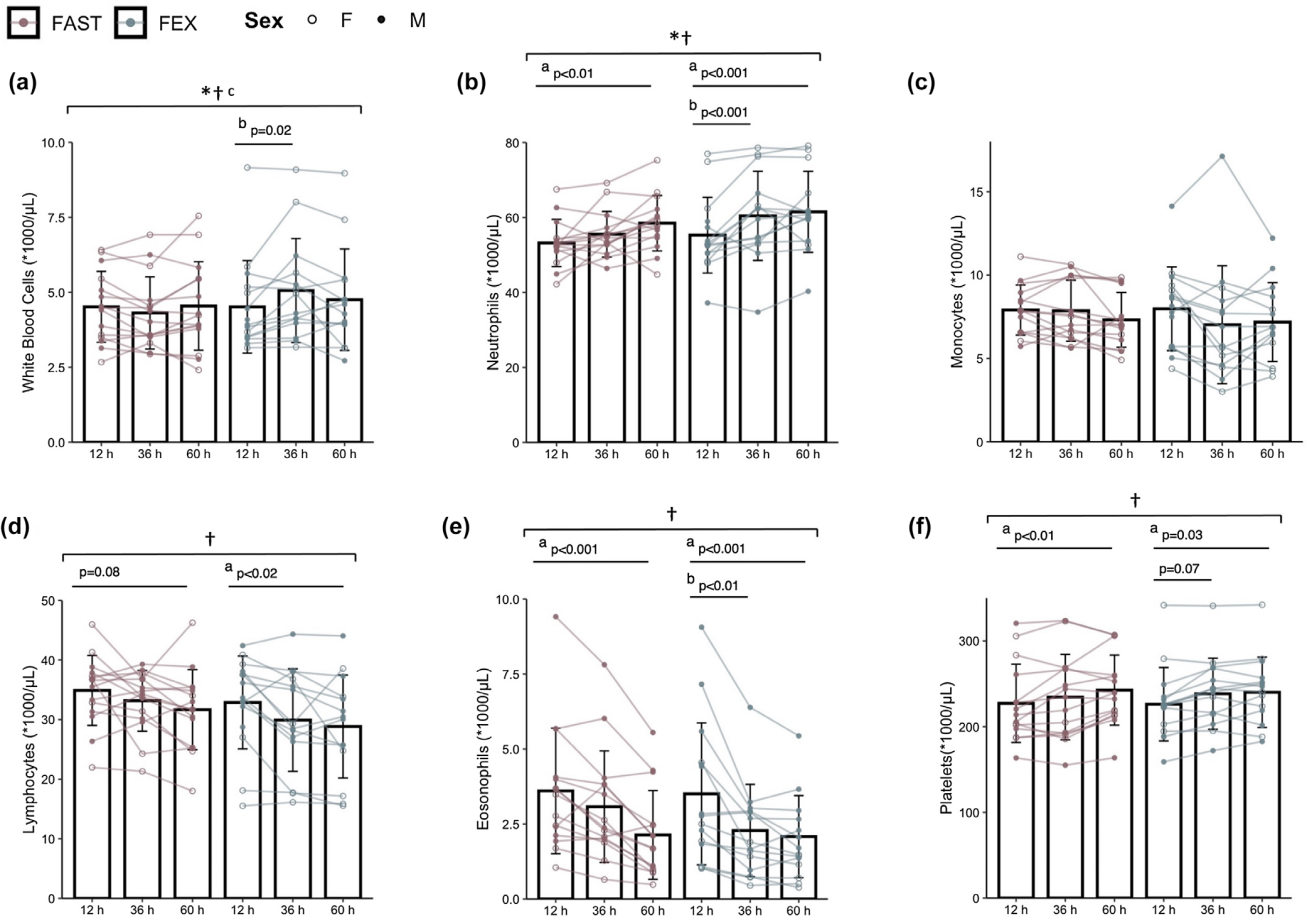
Changes in total white blood cells (a), neutrophils (b), lymphocytes (c), monocytes (d), eosinophils (e), and platelets (f) during each experimental condition. * Condition × Time Interaction, *p* < 0.05; ^†^Main effect of time, *p* < 0.05; ^a^12 h versus 60 h, *p* < 0.05; ^b^12 h versus 36 h, *p* < 0.05; ^c^FAST versus FEX at 36 h, *p* < 0.05; ^d^FAST versus FEX at 60 h, *p* < 0.05. Data are disaggregated by sex with open circles (_°_) indicating female participants and closed circles (•) indicating male participants.

Changes in total white blood cells, leukocyte subpopulations, and platelets are shown in Figure 2a–f. There was a significant condition*time interaction for total white blood cells (*p* < 0.01) with an increase from baseline to 36 h in FEX only (*p* = 0.02). A significant condition*time interaction effect was also apparent for neutrophils (*p* = 0.04). While neutrophils increased from baseline to 60 h in both FAST (*p* < 0.01) and FEX (*p* < 0.001), an increase at 36 h was apparent in FEX only (*p* < 0.001). Significant main effects of time were apparent for eosinophils (*p* < 0.001), platelets (*p* < 0.001), and lymphocytes (*p* < 0.01). Eosinophils decreased from baseline to 60 h in both conditions (both *p* < 0.001), with a more rapid decrease observed in FEX at 36 h (*p* < 0.01). Similarly, platelets increased from baseline to 60 h in both FAST (*p* < 0.01) and FEX (*p* = 0.03), with a tendency towards a more rapid increase observed in FEX at 36 h (*p* = 0.07). Lymphocytes decreased from baseline to 60 h in FEX only (*p* = 0.02), although the reduction in lymphocytes at 60 h in FAST also approached statistical significance (*p* = 0.08).

### 3.4 Plasma cytokines

**FIGURE 3:**
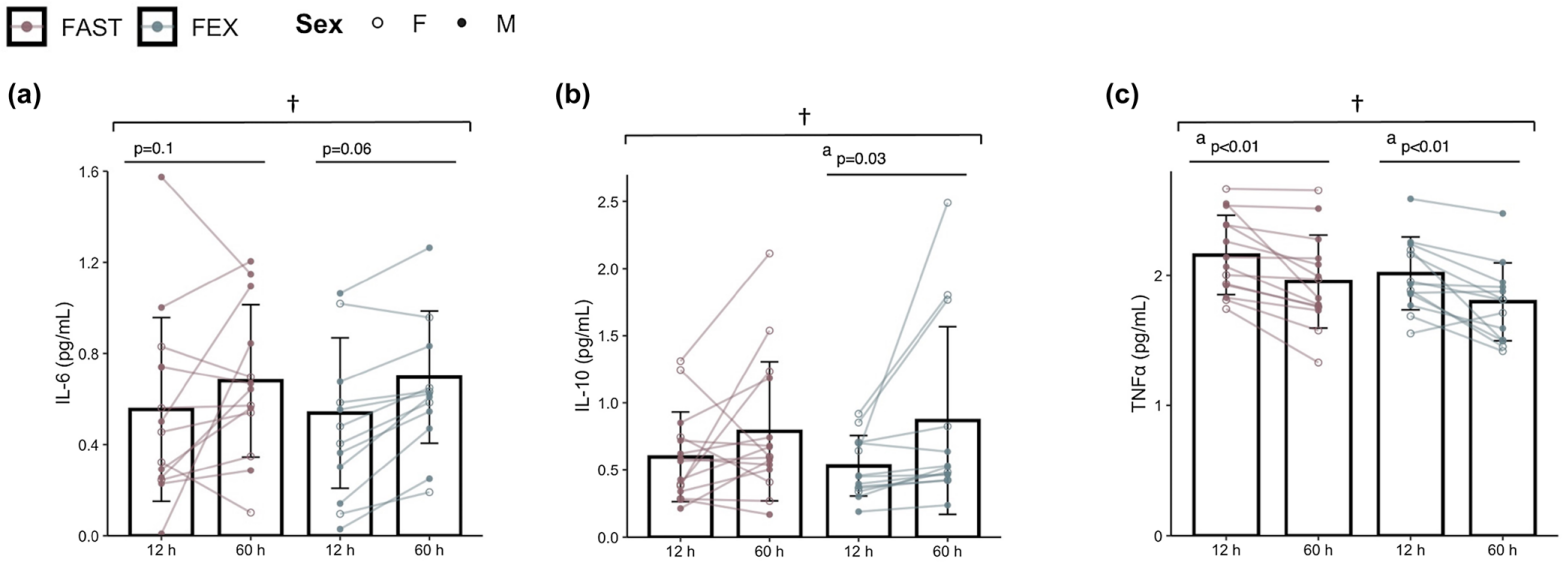
Changes in plasma interleukin (IL)‐6 (a), IL‐10 (b), and tumor necrosis factor‐alpha (TNF‐⍺; c) during each experimental condition. * Condition × Time Interaction, *p* < 0.05; ^†^Main effect of time, *p* < 0.05; ^a^12 h versus 60 h, *p* < 0.05; ^b^12 h versus 36 h, *p* < 0.05; ^c^FAST versus FEX at 36 h, *p* < 0.05; ^d^FAST versus FEX at 60 h, *p* < 0.05. Data are disaggregated by sex, with open circles (_°_) indicating female participants and closed circles (•) indicating male participants.

Changes in plasma IL‐6, IL‐10, and TNF‐⍺ concentrations are shown in Figure 3a–c. Significant main effects of time were observed for plasma IL‐6 (*p* = 0.02), IL‐10 (*p* < 0.01), and TNF‐⍺ (*p* < 0.01). Plasma IL‐10 concentrations increased from baseline to 60 h in FEX (*p* = 0.03) whereas TNF‐⍺ decreased in both conditions (*p* < 0.01). The increase in IL‐6 from baseline to 60 h approached statistical significance in FAST (*p* = 0.1) and FEX (*p* = 0.06). No significant condition*time interaction effects were apparent for any cytokine (*p* > 0.4). Plasma TNF‐⍺ concentrations decreased from baseline to 60 h in both conditions (both *p* > 0.01).

## 4 DISCUSSION

The present study determined the effect of a 60‐h fast alone or combined with a prolonged exercise bout on plasma cytokine and leukocyte concentrations, allowing us to investigate the interaction between altered whole‐body metabolism and immunological processes. A 60‐h fast lowered plasma glucose and insulin, increased ketones, and increased whole‐body fat oxidation with accelerated changes in glucose and BHB with the addition of exercise. These fasting‐induced metabolic changes were accompanied by reduced plasma concentrations of the pro‐inflammatory cytokine TNF‐α and increased plasma concentrations of the anti‐inflammatory cytokine IL‐10 (with a similar tendency for IL‐6) supporting an overall anti‐inflammatory effect of short‐term fasting. Circulating cytokine concentrations were not modulated by the addition of exercise despite more rapid decreases in plasma glucose and enhanced ketogenesis in the combined fasting and exercise condition. No changes in total leukocytes, and only modest changes in certain leukocyte subpopulations were apparent at 60 h in either condition, suggesting that an acute 60‐h fast does not appear to compromise immune function.

Studies in preclinical models highlight a potential anti‐inflammatory effect of short‐term fasting, as evidenced by reductions in circulating monocytes (Jordan et al., 2019), pro‐inflammatory cytokine concentration in plasma and adipose tissue (Speaker et al., 2016), and pro‐inflammatory gene expression (Jordan et al., 2019). However, evidence in humans is conflicting and comparatively limited. Evidence from chronic intermittent or religious fasting has suggested a shift towards a more anti‐inflammatory cytokine profile (Wang et al., 2020; Zouhal et al., 2020), but the associated changes in overall energy intake and body composition make it difficult to isolate the effects of fasting per se on inflammatory processes. Despite the potential for the altered metabolic state associated with short‐term fasting to induce favorable shifts in immune cell phenotype and function that suppress inflammatory signaling (Ménégaut et al., 2017), some evidence in humans suggests an exacerbated pro‐inflammatory cytokine profile in response to prolonged (36 h to 10 days) fasting (Fazeli et al., 2020; Mooren et al., 2020). Our observation of reduced circulating TNF‐α following a 60‐h fast both with and without exercise, as well as an increase in circulating concentrations of the prototypical anti‐inflammatory cytokine IL‐10 with combined fasting and exercise, supports the notion that short‐term fasting may suppress inflammatory processes. TNF‐α release from adipose tissue macrophages is a characteristic feature of obesity‐related chronic inflammation and has been implicated in the pathogenesis of insulin resistance and metabolic syndrome (Hotamisligil et al., 1995). As M1‐type macrophages mediate the release of pro‐inflammatory factors including TNF‐α (Odegaard & Chawla, 2011), the potential for fasting‐induced upregulation of fatty acid oxidation to induce a shift away from proliferation of M1 and towards M2‐type macrophages could explain the observed reduction in circulating TNF‐α.

The increase in circulating IL‐10 observed in FEX also supports an anti‐inflammatory effect of combined fasting and exercise. IL‐10 is secreted by various immune cell types and exerts anti‐inflammatory effects through the inhibition of pro‐inflammatory cytokine synthesis, as well as suppression of T‐cell proliferation (Saraiva et al., 2020). Inhibition of glycolysis as well as upregulated fatty acid metabolism in activated macrophages can enhance IL‐10 secretion (Saraiva et al., 2020), representing a potential mechanistic link between fasting‐induced alterations in whole‐body metabolism and the observed increase in circulating IL‐10. Acute exercise also increases IL‐10 secretion, an effect primarily mediated through the release of IL‐6 from contracting skeletal muscle (Islam et al., 2021; Pedersen & Febbraio, 2008). However, it has previously been reported that circulating IL‐10 levels return to baseline 24 h after a marathon run (de Sousa et al., 2021), suggesting our observation of a persistent elevation in IL‐10 48‐h post‐exercise is likely attributable to the amplified metabolic stress of combined fasting and exercise, rather than an isolated effect of prolonged strenuous exercise. The similar, albeit not statistically significant, increase in IL‐6 also supports a favorable effect of fasting on inflammatory processes. Although IL‐6 is traditionally viewed as a pro‐inflammatory cytokine (Scheller et al., 2011), the specific cell signaling properties of IL‐6 are context‐specific and may be partially dependent on cell type (Scheller et al., 2011). Specifically, the release of IL‐6 from skeletal muscle cells has been linked to anti‐inflammatory effects (Mauer et al., 2014). This is evidenced by the marked increase in IL‐6 following prolonged exercise, which elicits anti‐inflammatory effects through the subsequent upregulation of IL‐10 and inhibition of pro‐inflammatory cytokine release (Pedersen & Febbraio, 2008). Similarly, fasting has been shown to induce increases in IL‐6 mRNA in muscle cells, but not hepatocytes or adipocytes in a mouse model (Wueest et al., 2014). Given its lipolytic properties, IL‐6 release may also play a role in metabolic regulation during fasting (Pedersen et al., 2003; Wueest et al., 2014).

Given the integration of metabolic and immunological processes, the possibility for changes in nutrient availability to affect the host response to invading pathogens is a significant area of interest. Reductions in circulating leukocyte subpopulations have been observed in response to fasting due to the redistribution of immune cells to the bone marrow and/or impaired mobilization into peripheral circulation (Janssen et al., 2023; Jordan et al., 2019), suggesting short‐term fasting could impair the immune response. Although we observed some alterations in leukocyte subpopulations, total white blood cell count remained unchanged in FAST and was slightly elevated relative to baseline at 36 h in FEX, suggesting overall maintenance (or enhancement) of immune function. The observed increase in neutrophils and decrease in eosinophils and lymphocytes mimics the transient changes in circulating leukocytes that would be expected following acute exercise (Peake et al., 2017; Spijkerman et al., 2021). However, these alterations are usually reversed within a few hours post‐exercise (Peake et al., 2017), indicating the observed changes in FEX are likely not a direct effect of exercise. It could be speculated, however, that the mechanisms underlying exercise‐induced changes in leukocyte subpopulations—specifically increased catecholamines and cortisol levels (Zahorec, 2021)—may also explain the similar alterations in circulating immune cells observed with prolonged fasting, which elicits a comparable hormonal response (Boyle et al., 1989).Along these lines, the accelerated response of some of these parameters in FEX could reflect the exacerbated metabolic stress and/or a synergistic effect of combined fasting and exercise on catecholamine and cortisol secretion. Although total leukocyte and monocyte counts remained stable across the 60 h fast in both conditions, it is possible that subtle shifts in leukocyte distributions seen (i.e., increased neutrophils, decreased eosinophils and lymphocytes)—combined with an overall anti‐inflammatory cytokine profile (discussed above)—could also be indicative of a transient immunosuppression. Future work should include additional indices of immune function to provide additional insights into immunological changes with prolonged fasting and/or exercise.

As anticipated, insulin and glucose were significantly decreased in both conditions, although the drop in blood glucose occurred earlier at 36 h in FEX. A decrease in RER was also observed in both conditions, indicating a shift towards greater fat oxidation with prolonged fasting. The addition of a prolonged exercise bout at the beginning of the fast accelerated the onset of ketogenesis, as indicated by a significant elevation in plasma BHB at 36 h in FEX; however, this magnitude of increase was not apparent until 60 h in FAST (and remained lower than FEX at this time‐point). The accelerated ketogenesis is likely owing to the more rapid depletion of muscle and hepatic glycogen stores, and associated reductions in blood glucose (Evans et al., 2017). As such, prolonged exercise has been shown to produce elevations in ketone bodies in the hours following exercise cessation (Schranner et al., 2020); these prolonged elevations have previously been shown to accelerate fasting‐induced ketone body production (Deru et al., 2021). Although combined fasting and exercise seemingly augmented ketone body production, this did not appear to influence inflammatory markers, as there were no significant between‐condition differences. However, the potential ability of prior exercise to augment the ketogenic effects of fasting may have implications for enhancing the health benefits attributable to fasting‐induced exposure to endogenous BHB—namely, enhanced metabolic and cognitive function, and reduced inflammation (Newman & Verdin, 2017). While previous studies have demonstrated conflicting results regarding the effect of fasting on energy expenditure, the slight increase in RMR after 60 h in both conditions aligns with findings from fasting protocols of similar duration (Mansell et al., 1990; Zauner et al., 2000). While our assessment of RMR via indirect calorimetry may be limited to some extent due to the relatively short duration of gas exchange measurement and the lack of a prior rest period (Compher et al., 2006), our observation of an increased metabolic rate following 60 h of fasting could reflect greater adrenergic stimulation, and/or the energetic cost of increased gluconeogenesis during fasting (Veldhorst et al., 2009).

A few limitations of the present study should be noted. First, we only measured circulating cytokine concentrations, which gives limited insight into cytokine action at the cellular level (Zauner et al., 2000). Given the pleiotropic nature of certain cytokines measured (e.g., IL‐6 and IL‐10), adequate assessment of cytokine action and/or the addition of immunophenotyping would allow for a more comprehensive understanding of the immunomodulatory effects of fasting, providing an important area for future work. Additionally, when measuring plasma cytokines, one cannot account for their site of release or mechanism of production. Although our model of combined fasting and exercise allows for mechanistic insight into the interaction between whole‐body metabolism and immunological processes, we cannot make any definitive takeaways as to the potential clinical applications of short‐term fasting based on the present findings. Specifically, the alterations in immunological and inflammatory markers observed in the present study represent the effects of fasting in isolation. An understanding of how refeeding after a fast also influences immunometabolic processes is warranted to comprehensively examine the therapeutic implications of short‐term fasting for combating chronic inflammation and improving various health parameters. It is also important to note that since plasma cytokine concentrations were only measured before and after the 60‐h fast, we may have missed potential accelerated increases as a result of the exercise bout that occurred earlier in the fast (i.e., at 36 h). Additionally, while we asked participants to match caffeine intake between conditions and prior to testing, allowing participants to consume caffeinated beverages throughout the fast could have impacted our results given the previously reported immunomodulatory effects of caffeine (Kovács et al., 2021). We also recognize that while we tested females during the same menstrual cycle between conditions, hormonal fluctuations throughout the menstrual cycle may have impacted our results. Finally, as our sample was comprised of healthy young adults, these findings cannot be extended to clinical populations, given the various immunological and metabolic defects present in chronic disease.

In conclusion, our results suggest alterations in whole‐body metabolism achieved through fasting with or without the addition of exercise support an anti‐inflammatory cytokine profile, without altering total leukocyte concentration. Future studies should implement more comprehensive measures examining cytokine action, gene expression, and immune cell phenotype and function to confirm these findings. Further investigation of the immunometabolic effects of short‐term fasting (and refeeding) in clinical populations to explore the therapeutic potential of short‐term fasting, as well as other physiological stimuli that produce similar alterations in whole‐body metabolism, is also needed.

## FUNDING INFORMATION

TDG was supported by a Killam Postdoctoral Fellowship. HI was supported by a Michael Smith Health Research BC Research Trainee Award.

## CONFLICT OF INTEREST STATEMENT

None.

## ETHICS STATEMENT

Verbal and written informed consent was obtained from all participants prior to data collection in accordance with the Declaration of Helsinki and the study was approved by UBC’s clinical research ethics board (H22‐01713).

## Data Availability

All data are available upon reasonable request made to the corresponding authors.
